# Early Detection of *Magnaporthe oryzae*-Infected Barley Leaves and Lesion Visualization Based on Hyperspectral Imaging

**DOI:** 10.3389/fpls.2018.01962

**Published:** 2019-01-15

**Authors:** Rui-Qing Zhou, Juan-Juan Jin, Qing-Mian Li, Zhen-Zhu Su, Xin-Jie Yu, Yu Tang, Shao-Ming Luo, Yong He, Xiao-Li Li

**Affiliations:** ^1^College of Biosystems Engineering and Food Science, Zhejiang University, Hangzhou, China; ^2^Zhejiang Machinery Industry Information Institute, Hangzhou, China; ^3^College of Agriculture and Biotechnology, Zhejiang University, Hangzhou, China; ^4^Ningbo Institute of Technology, Zhejiang University, Ningbo, China; ^5^College of Automation, Zhongkai University of Agriculture and Engineering, Guangzhou, China

**Keywords:** barley, *Magnaporthe oryzae*, spectral unmixing analysis, infection period identification, lesion visualization

## Abstract

Early detection of foliar diseases is vital to the management of plant disease, since these pathogens hinder crop productivity worldwide. This research applied hyperspectral imaging (HSI) technology to early detection of *Magnaporthe oryzae*-infected barley leaves at four consecutive infection periods. The averaged spectra were used to identify the infection periods of the samples. Additionally, principal component analysis (PCA), spectral unmixing analysis and spectral angle mapping (SAM) were adopted to locate the lesion sites. The results indicated that linear discriminant analysis (LDA) coupled with competitive adaptive reweighted sampling (CARS) achieved over 98% classification accuracy and successfully identified the infected samples 24 h after inoculation. Importantly, spectral unmixing analysis was able to reveal the lesion regions within 24 h after inoculation, and the resulting visualization of host–pathogen interactions was interpretable. Therefore, HSI combined with analysis by those methods would be a promising tool for both early infection period identification and lesion visualization, which would greatly improve plant disease management.

## Introduction

As the global population is projected to exceed 9 billion by 2050, large-scale crop production is needed to meet the growing demand for food and animal feed (Borlaug, 2000). However, crop yields are inevitably limited by plant stresses, especially biotic factors like fungal disease. Barley (*Hordeum vulgare* L.) is the world’s fourth largest cereal crop, and plays a major role in global crop production (Byungkee and Stevene, 2008). Barley is a high-quality feed for animal husbandry and aquaculture. Moreover, it is an important raw material for the brewing industry (Linko et al., 1998; Byungkee and Stevene, 2008). In order to satisfy the demand for higher levels of quality in beer and other agricultural and food products, more attention should be paid to the breeding and cultivation of barley as a raw material (Porker et al., 2016). Foliar diseases can destroy leaf structures, impair photosynthesis, and accelerate senescence as well as apoptosis of leaves, which results in large reduction of the crop yield and increasing economic losses (Yu et al., 2013).

*Magnaporthe oryzae* (*M. oryzae*) B.C. Couch [formerly *Magnaporthe grisea* (T.T. Hebert) M.E. Barr], a filamentous phytopathogenic fungus, is a causal agent of blast diseases in many cereal crops including barley (Guo et al., 2017). The infection process of *M. oryzae* has been reported to involve the invasion of barley leaves by penetration of epidermal cells (Ulferts et al., 2015). A specialized dome-shaped cell called the appressorium generates strong biological turgor pressure to break through the host cuticle (Talbot, 2003). Then, the invasive hyphae of *M. oryzae* grow in the epidermal cells, enlarging the invasion (Ulferts et al., 2015). Cereal leaf diseases such as *M. oryzae* frequently infect barley plants and seriously affect the crop quality and economic value of malting barley (Yu et al., 2013). Here, cultivation of resistant varieties and application of fungicides are the two main methods to reduce the loss caused by barley leaf diseases (Walters et al., 2012). However, the phenotyping process, i.e., analysis of the expression of the genome in a particular environment, is time-consuming, laborious and costly (An et al., 2016). Therefore, the development of an easy and fast method for early detection would be a vital asset in the management of barley diseases like *M. oryzae*. On the one hand, early detection would help crop managers to ensure the timely application of fungicides, and on the other hand, better phenotyping methods would have the potential to improve the breeding process (Kuska et al., 2015).

Traditional molecular methods to detect foliar diseases are based on immunological techniques including enzyme-linked immunosorbent assays (ELISA) (Harrison et al., 2010) and DNA-based techniques such as polymerase chain reaction (PCR) (Glen et al., 2007; Sun et al., 2015). Although these standard methods are accurate, they are not reliable enough at the asymptomatic stage (Martinelli et al., 2015). Moreover, the intricate sample preparation, long detection time, and lack of phenotyping information limit the feasibility of these methods in large-scale field detection and *in situ* analysis. Some innovative approaches using optical imaging sensors to achieve early identification and detection have been reported, such as RGB (Sannakki et al., 2011), fluorescence (Chaerle et al., 2007) and hyperspectral imaging (HSI) technology (Xie et al., 2015). Among these methods, HSI is one of the most promising techniques for foliar disease assessment and monitoring (Mahlein et al., 2012a; Zhao et al., 2016). HSI can detect both spectral and spatial information with high resolution, which enables the improvement of disease detection through a better observation of host-pathogen interactions (Bock et al., 2010; Kong et al., 2018). The reflectance spectra of each pixel in the leaves display multiple interactions between the irradiation and the biophysical and biochemical traits of the leaves (Mahlein et al., 2012b). Leaf pigments are the main factors influencing reflectance spectra in the visible region, while certain tissue structures and components (e.g., water, proteins, and fatty acids) are the main factors affecting the spectra in the near-infrared (NIR) region (Kuska et al., 2015). Therefore, changes in the pigments or tissue structure of leaves caused by fungal disease can be detected early through analyzing the spectra.

As HSI contains abundant spectral and spatial information, processing the acquired data depends on the problem being solved, such as detecting the presence of disease and early stage detection of disease symptoms (Singh et al., 2016). To ensure early detection of host-pathogen interaction, visualization of sample is important, so that every single pixel should be analyzed. General data processing reduces spectral dimensions for easier analysis. Extraction of spectral features usually includes vegetation indices (Lu et al., 2018), disease indices (Mahlein et al., 2013) and a subset of characteristic wavelengths which could be selected by some methods like stepwise discriminant analysis (Yeh et al., 2016). Although extracted features would simplify the data and make sample visualization easier, some useful information would inevitably go missing, since they are part of full spectrum data. For full spectrum analysis, some statistical methods like principal component analysis (PCA) (Bagnasco et al., 2015) and cluster analysis methods based on distance like *K*-means (Leucker et al., 2016) and spectral angles like spectral angle mapping (SAM) (Mahlein et al., 2012b) were commonly used. However, the statistical methods extract the abstract feature contained in full spectrum analysis, which cannot be interpreted clearly in spectral curves. On the other hand, cluster analysis assigns each pixel to a cluster, which cannot reflect the gradual process of fungal infection if the number of clusters is too low. In this study, we try to track the changes of reflectance spectra of barley during fungal infection, and divide the lesion spectra into different clusters, corresponding to different infection periods. However, the spectra of pixels in the host-pathogen interaction regions contain information from different infection periods because infection is a gradual process, which cannot be accurately assigned to a single period. Spectral unmixing, a prominent method for mining spectral information, has been proposed to solve this problem (Keshava and Mustard, 2002). Spectral unmixing introduced here is derived from remote sensing, which addresses similar problems by extracting specific component information from the mixed spectra, except that the mixture change from different components to different infection periods. Spectral unmixing consists of two parts (Ma et al., 2014): part one, extracting the component spectra (endmembers) in the hyperspectral images, which is unsupervised and corresponding to different infection periods; part two, decomposing mixed spectra and deducing the spatial distributions (abundances), which represent the ratio of each infection period in pixels rather than assign pixels to a single period. Therefore, spectral unmixing would be suitable to visualize the gradual process of infection from the infection center to the edge as the pixels in those regions are mixed with different infection periods.

In this research, the objective was to explore the feasibility of HSI for early detection of barley disease and visualization of lesion regions. The specific objectives were: (1) to describe the spectral characteristics of barley leaves infected by *M. oryzae* in consecutive infection periods, (2) to non-destructively achieve the early identification of infectious degree and period, and (3) to accomplish the early visualization of lesion regions, before the appearance of visual symptoms, based on spectral unmixing analysis.

## Materials and Methods

**FIGURE 1:**
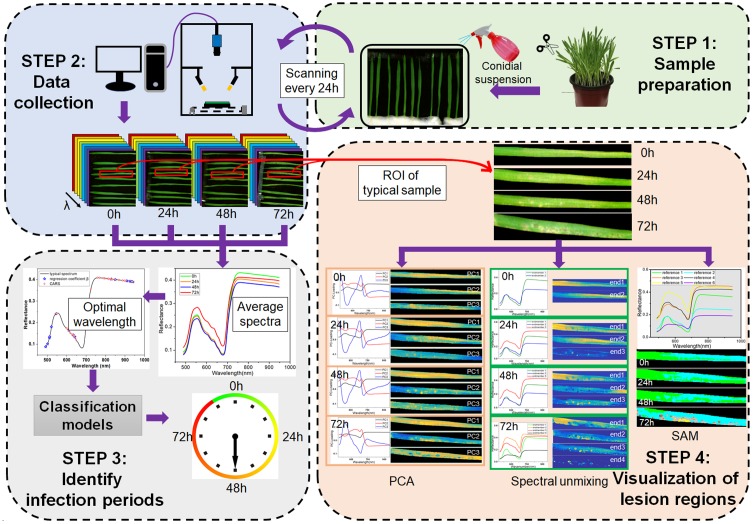
Framework of early detection of barley leaves infected by *Magnaporthe oryzae* and visualization of lesion regions.

The framework of this study is shown in Figure 1, which consists of four main steps. The first step was sample preparation, including barley cultivation and pathogen inoculation. The second step was data collection, i.e., the consecutive scanning of barley leaves every 24 h. The third step was identification of the infection periods based on applying the classification algorithms to the averaged spectra at optimal wavelengths. The fourth step was visualization of lesion regions using PCA, spectral unmixing and SAM.

### Plant Cultivation and Pathogen Inoculation

The seeds of barley plants (*Hordeum vulgare* L., cv. Golden Promise) were used in this study. The plants were grown in a greenhouse at 23/20°C (day/night), 60% relative humidity for 7 days. The primary leaves of 38 barley plants (length about 10 cm) were detached and transferred to a moistened chamber. *M. oryzae* Guy11 (wild-type strain) was grown at 25°C on complete medium [10 g of D-glucose, 1 g of Yeast extract, 2 g of Peptone, 1 g of Casamino acids, 6 g of NaNO_3_, 1.52 g of KH_2_PO_4_, 0.52 g of KCl, 0.52 g of MgSO_4_⋅7H_2_O, 0.1%(v/v) Vitamin solution (100 ml 1000 × Vitamin solution includes 0.01 g Biotin, 0.01 g Pyridoxin, 0.01 g Thiamine, 0.01 g Riboflavin, 0.01 g p-aminobenzoic acid, and 0.01 g Nicotinic acid], 0.1%(v/v) Trace elements (100 ml 1000 × Trace elements includes 2.2 g ZnSO_4_⋅7H_2_O, 1.1 g H_3_BO_3_, 0.5 g MnCl_2_⋅4H_2_O, 0.5 g FeSO_4_⋅7H_2_O, 0.17 g CoCl_2_⋅6H_2_O, 0.16 g CuSO_4_⋅5H_2_O, 0.15 g NaMoO_4_⋅5H_2_O, 5 g Na_4_EDTA, and 15 g of agar per liter, pH 6.5) for 14 days. After that, conidial suspension (1 × 10^6^ spores/ml) was sprayed onto the barley leaves and incubated in a controlled environment (temperature of 25°C and 80% relative humidity) for 72 h.

### Hyperspectral Imaging System and Data Acquisition

A line-scanning HSI system covering the visible and NIR wavelengths (380–1023 nm with 2.8 nm spectral resolution) was used in this study. It consists of a lens (OLE-23) mounted on an imaging spectrograph (V10E-QE, Specim, Finland), a conveyer belt driven by a stepper motor (IRCP0076, Isuzu Optics Corp., Taiwan, China), two 150 W line tungsten halogen lamps (DCRIII, Schott Glass Co., Elmsford, NY, United States) and a computer. The HSI data were obtained by line scanning using a camera (C8484–05, Hamamatsu City, Japan).

All samples were put on a blackboard with low reflectivity, and both ends were fixed with tape to reduce the influence of unevenness. Before inoculation, all 38 samples were scanned as healthy samples. To consecutively monitor the infection process, each inoculated leaf was, respectively, scanned at 24, 48, and 72 h, and immediately put back into the moistened chamber after each data acquisition. Uniform acquisition parameters were set for each scan. The exposure time was 0.03 s, the moving speed was 3.2 mm/s and the vertical distance from samples to the lens was 33 cm. Thus, a total of 152 hyperspectral cube data points were obtained by scanning the 38 samples at four infection periods.

### Pre-processing of Hyperspectral Images

The raw hyperspectral images were firstly corrected using white and dark reference images following a previously reported procedure (Xie et al., 2015). After that, the background of the corrected hyperspectral images was masked. The averaged spectra of each barley leaf were obtained, and the hyperspectral cube data were also recorded for further analysis. Due to the large noise at both ends of the spectra, only the wavelengths between 485 and 945 nm were selected. Furthermore, all the averaged spectra and the spectra of all pixels were smoothed by moving average filtering, and the span for the moving average was set to 7.

### Classification of Infectious Degree and Stages

#### Classification Models

To establish an efficient and stable classification model, the 152 samples were divided into a calibration set with 100 samples and a prediction set with 52 samples. Then, in order to identify the healthy leaves as well as the different infection periods of the inoculated leaves, four well-known classification algorithms including linear discriminant analysis (LDA) (Kak and Martínez, 2001), partial least square-discriminant analysis (PLS-DA) (Barker and Rayens, 2003), *k*-nearest neighbor (KNN) (Ni et al., 2009) and soft independent modeling of class analogy (SIMCA) (Maesschalck et al., 1999) were applied to establish classification models based on the averaged spectra.

Linear discriminant analysis, which is widely used for dimensionality reduction and classification, projects high-dimensional data onto a low-dimensional space to promote class separability. The optimal projection in classical LDA is obtained by maximizing the distance among different classes and minimizing the distance within the same class. PLS-DA is a widely used method for supervised pattern recognition, and is a variant of partial least square regression (PLSR) with the input of category value instead of response value. PLS-DA linearly transforms the spectral data into new latent variables (LVs), such that the first few LVs carry the main spectral information. The outputs of PLS-DA values are assigned to the closest categories. KNN firstly calculates the distance between the input sample and all the samples in the training set. Then, the category with the largest number of members among the *k*-nearest neighbors of the test sample is considered as the category of the sample. In this study, the optimal value of *k* was decided by 10-fold cross-validation in the training set. SIMCA is another widely used supervised pattern recognition method, based on PCA. SIMCA performs PCA on each category of the samples, then analyzes the unknown samples using the various PCA models one by one. Finally, SIMCA classifies the unknown samples based on the spectral residuals.

#### Optimal Wavelength Selection

The acquired spectra consist of hundreds of wavelength variables, which may contain many non-informative variables and some that are collinear (Zhang et al., 2016). Besides, an excessively large number of wavelength variables would make the training models unstable and difficult to interpret. The host–pathogen interaction changes the external and internal traits of the barley leaves. Therefore, the optimal selection of wavelengths from the acquired dataset would include only those that elucidate these changes to the infected samples. In this study, two methods – the regression coefficient (β) of PLSR (Mehmood et al., 2012) and competitive adaptive reweighted sampling (CARS) (Li et al., 2009) – were used to extract the optimal wavelengths. β is a measure of correlation between each spectral variable and the category value. Therefore, variables with high absolute value were retained as the optimal wavelengths. The CARS algorithm is based on the principle of “survival of the fittest” in Darwin’s theory of evolution, and uses an exponentially decreasing function and adaptive reweighting sampling to select the variables with the largest absolute values of the regression coefficient.

### Infection Spot Identification and Visualization

As the host–pathogen interaction sites are distributed sparsely, the averaged spectra often obscure the spectral information of early lesion regions. Fortunately, HSI can detect both spectral and spatial information with high resolution, which enables the early identification of lesions. Since the modifications in the pigments or tissue structures of leaves caused by fungal disease can affect the spectra in those pixels, the lesion regions can be distinguished from the healthy parts based on their spectral characteristics. In this study PCA, spectral unmixing analysis and SAM were applied to achieve the visualization.

Generally, the score images obtained from PCA models can be used to visualize different components of the tested leaf according to the variance information covered by each score image (Kamruzzaman et al., 2012). In this study, the first three principal components (PCs), which accounted for more than 99.5% of the variance of the data, were used to generate the score images. Spectral unmixing is the procedure of decomposing the mixed spectrum of each pixel into its constituent endmembers (pure components) and the abundances (i.e., the spatial distributions) of the endmembers (Keshava and Mustard, 2002). In this study, vertex component analysis (VCA) as an unsupervised approach was applied to extract the endmembers based on the convex geometry theory (Nascimento and Dias, 2005). After that, non-negative constrained least-squares was performed on the mixed spectra of the pixels to calculate the abundance of each pixel. Some studies have shown that the results of spectral unmixing resuanalysis are relatively easy to interpret, as the method obtains more meaningful spectra and clearer distribution images (Li et al., 2017). SAM is a well-known spectral classification approach, which calculates the “similarity” of each pixel from its spectral angles, i.e., the angles between its spectrum and a set of reference spectra (Mahlein et al., 2012b). According to its similarity, each pixel is assigned to several known categories. However, the reference spectra must be carefully chosen to represent unique signatures before calculating the similarity. In this research, the reference spectra were chosen based on the endmembers extracted by VCA.

### Software and Model Evaluation

In this study, the hyperspectral images were masked using the software ENVI 5.1 (ITT Visual Information Solutions, Boulder, United States). The spectral smoothing, classification models, optimal wavelength selection and visualization of the samples were all conducted using MATLAB (R2016a) software (The MathWorks, Inc., Natick, MA, United States). The classification performances were evaluated by two criteria: the classification accuracy in the calibration and prediction sets, and the calculated receiver operating characteristic (ROC) curves. The performance of lesion visualization was evaluated by parallel comparisons with RGB images and self-comparisons in time series. On the basis of the continuity of infection, the spots of infection would remain for the next period. We judged the accuracy of the spot recognition by comparing it with next period, when the RGB images cannot show the exact position.

## Results

### Spectral Characteristics of Diseased and Healthy Leaves

**FIGURE 2:**
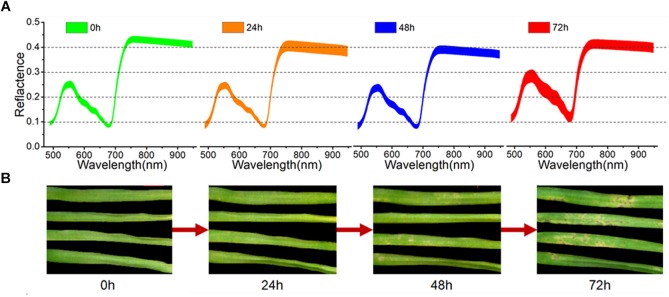
Averaged spectra, with standard deviation, of all samples **(A)** and RGB images of four representative samples **(B)** at four periods.

As seen in Figure 2B, the first symptoms of *M. oryzae* appeared as a series of light-yellow spots at 48 h of infection. With ongoing infection, these spots spread rapidly in the following 24 h, and many spots turned dark, allowing them to be easily recognized at 72 h. However, after only 24 h, it was barely possible to identify the successfully infected leaves from the RGB images. The averaged spectra, with standard deviation, of the healthy leaves and three periods of inoculated leaves are shown in Figure 2A. During the first 48 h of infection, the spectral curves showed a continuous decline in the green region (540–560 nm) and the NIR region (750–950 nm). In contrast, there was a significant rise in the visible region of the spectra at 72 h, which reflected a wide degree of change in pigments and structure.

**FIGURE 3:**
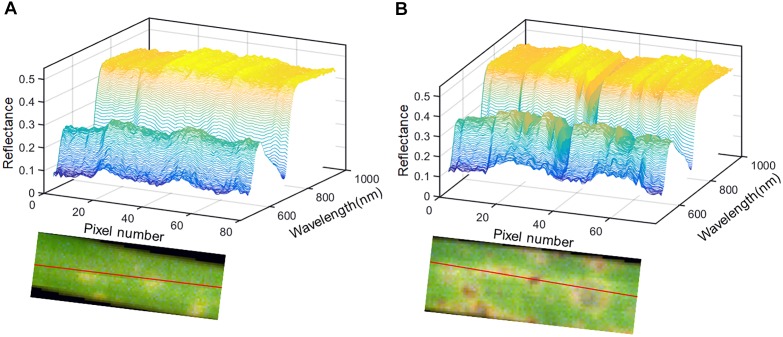
Pixel-wise reflectance from transects through light-yellow spot **(A)** and dark spot **(B)**.

Here, the spectral reflectance signatures were extracted pixel-wise from both healthy tissue and lesion regions (including light-yellow spot and dark spot symptoms), and are shown in Figure 3. As shown in Figure 3A, the spectra of healthy leaf tissue at the edge of the leaf were almost homogeneous. However, the existence of light-yellow spots significantly changed the trends of the spectra. In detail, the reflectance spectra of the spot centers were more intense in both the visible and NIR regions than the spectra of the healthy parts. Figure 3B clearly shows that the reflectance spectral intensity of the dark lesions drastically dropped in the visible and NIR regions (−26% in 550 nm and −15% in 800 nm). In our samples, the dark spots usually appeared in the center of light-yellow spots, and were considered as an advanced symptom of light-yellow spots. Slices of tissue structures obtained during fungal infection were observed by optical microscopy. The microscopic images corresponding to healthy tissue, light-yellow spots and dark spots are shown in Supplementary Figure S1 of the Supporting Information. The microscopic image of light-yellow spots revealed collapsed cells with clear decomposition of the pigment compared with the healthy tissue. The microscopic image of dark spots showed cells in a more serious state of collapse, which confirmed the development of the disease symptoms.

### Discriminant Models Using Full Spectra and Optimal Variables

In this study, PLS-DA, KNN, and SIMCA models were built using the full averaged spectra to identify the healthy leaves and the infected leaves at different inoculation periods. One healthy period (0 h) and three infection periods (24, 48, and 72 h) were defined, requiring a total of four categories to be classified. Through cross-validation, the optimal number of LVs in PLS-DA was chosen as 17 and the *k*-value in KNN was chosen as 4. The PLS-DA model achieved 100% classification accuracy of the calibration set, but the accuracy of the prediction set was only 88.5%. This indicated that the generalizability of the PLS-DA model to the full spectra was relatively low. The SIMCA model returned 99 and 90.4% classification accuracy of the calibration and prediction sets, respectively, which was a more robust performance. However, the performance of the KNN model was much worse than the other two with only 74 and 67.3% classification accuracy of the calibration and prediction sets. In summary, both PLS-DA and SIMCA could be used to build models to discriminate adequately among the four categories.

**FIGURE 4:**
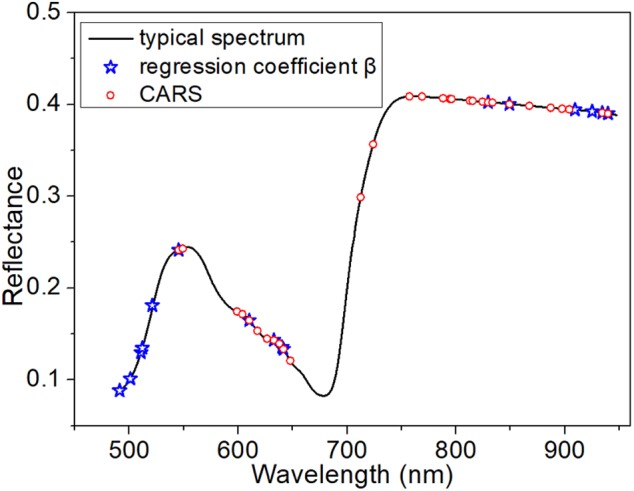
Distribution of optimal variables extracted from β of PLSR and from CARS.

The full averaged spectra in the spectral range from 485 to 945 nm contained more than 360 variables. Therefore, selection of the optimal variables was necessary to build a more informative model. When using PLSR, in which the threshold of the absolute value of β was set to be >150, 16 wavelength variables were obtained (indicated by blue stars in Figure 4). In contrast, CARS, based on an exponentially decreasing function and adaptive reweighting sampling, returned 30 optimal wavelengths (indicated by red circles in Figure 4).

Here, having defined the optimal variables, LDA was used to build a model based on those variables. Note that LDA requires a larger number of samples than variables. As shown in Supplementary Table S1 of the Supporting Information, LDA was capable of classification using both groups of variables. In summary, the variables extracted by CARS were more suitable for these samples. LDA and SIMCA both achieved more than 97% classification accuracy of the calibration set and over 80% classification accuracy of the prediction set with the variables extracted from CARS. This is because the reflectance of the characteristic variables extracted by CARS was very sensitive to the progress of infection, as seen in Figure 2A. However, PLS-DA and KNN returned poor classification accuracy in the prediction set. The best classification accuracy was achieved by the combination of LDA and the 30 variables extracted by CARS, with 100% classification accuracy of the calibration set and 98.1% classification accuracy of the prediction set. The ROC curve further verified the good performance of this classification model, as shown in Supplementary Figure S2 of the Supporting Information.

### Visualization of Lesion Regions at Different Infection Times

**FIGURE 5:**
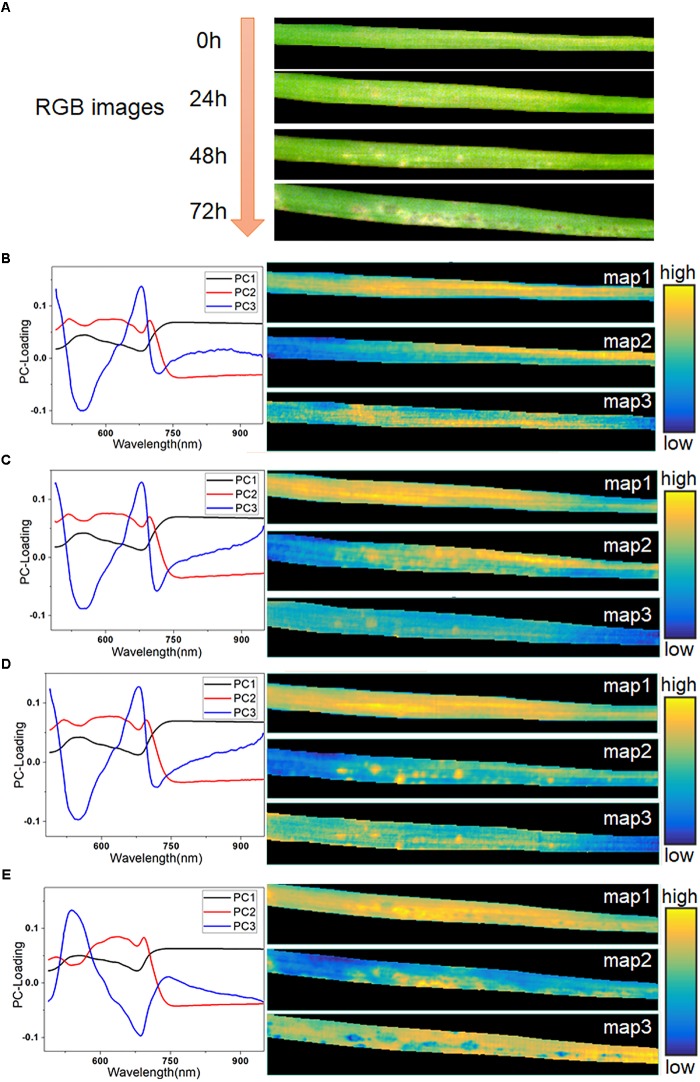
RGB images of typical leaf sample in four infection periods **(A)**, PC-loading curves and corresponding score maps of all four infection periods at 0 h **(B)**, 24 h **(C)**, 48 h **(D)**, and 72 h **(E)**.

The averaged spectra obscured the early spectral information of the host-pathogen interaction sites. Therefore, it was necessary to extract the spatial information from HSI in order to identify lesion regions before visual symptoms appeared. In this study, hyperspectral images of typical samples at consecutive infection times of 0, 24, 48, and 72 h were used for imaging analysis. The RGB images of these four stages are shown in Figure 5A. The initial symptom was light-yellow spots, which could be seen as early as 48 h. Then, the infection progressed rapidly in the following 24 h. At 72 h, some light-yellow spots turned dark, and the infected region began to spread. Meanwhile, with the decomposition of chlorophyll, large yellow patches appeared in the lesion regions.

Firstly, PCA was applied to analyze the hyperspectral images. Three score maps, corresponding to the first three PCs, were used to display different characteristic areas of the samples, since these three components had explained more than 99.5% of the variance of the data. As seen in Figures 5B–E, the curve of PC 1 loading closely resembled the spectra of the leaf in Figure 2A, as it accounted for the largest part of the spectral information. The score map of PC1 contained hits from all regions of the leaf except the edges. Most of the information in PC1 was common between each pixel, so it offered little value for further identification. At 0 h (Figure 5B), the score maps embodied the differences of reflectance caused by the differences of chemical composition in different parts of the leaf. The curves of PC2 and PC3 were similar, as shown in Figures 5C,D. However, their score maps were different. The score map of PC3 at 24 h showed a faint but recognizable image of the lesion regions, which demonstrated the feasibility of PCA analysis for early identification. At 48 h, the presence of infection spots was marked on the score map of PC2 in bright yellow, matching the light-yellow spots in the RGB image at 48 h. The symptoms of pathogen infection became obvious at 72 h, and showed complicated features, as also seen in the RGB image. The value of PC2 in the final PC-loading image at 72 h was lower than those in the first three images at 450–600 nm. Moreover, the curve of PC3 at 72 h (Figure 5E) showed completely opposite features compared with the PC3 curves in the previous periods. Here, the score map of PC2 at 72 h mainly reflected the light-yellow regions with high spectral reflectance. Meanwhile, the score map of PC3 at 72 h also included a number of dark blue spots.

**FIGURE 6:**
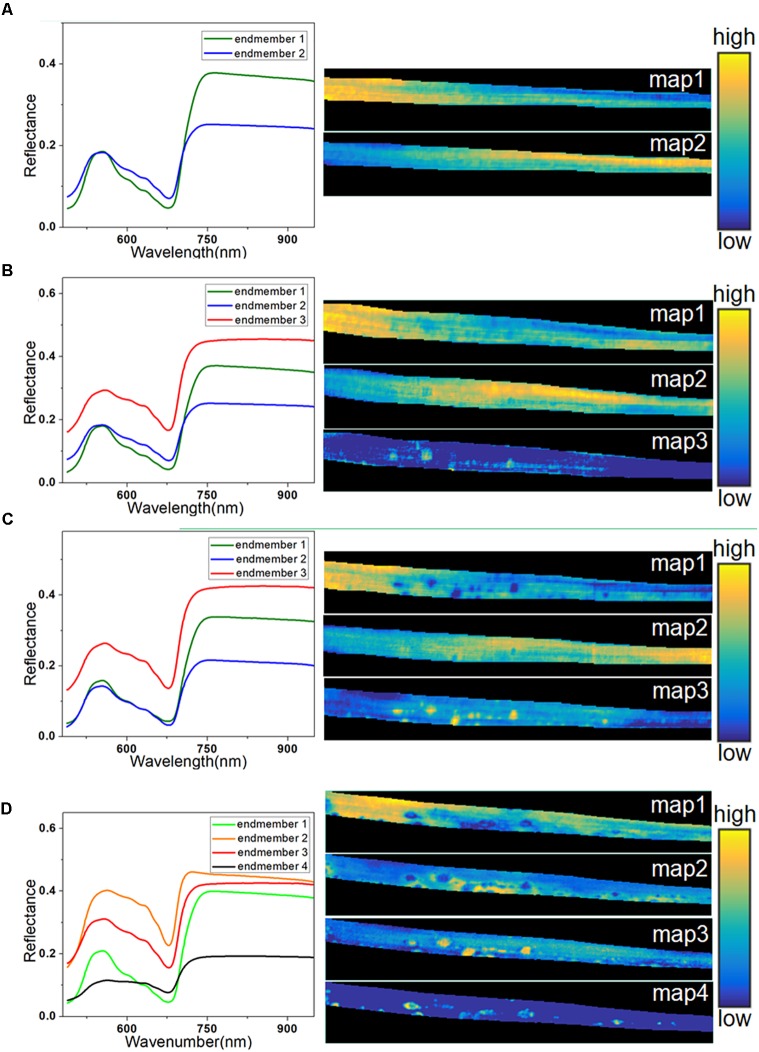
Endmembers and corresponding abundance maps of typical leaf sample in four infection periods at 0 h **(A)**, 24 h **(B)**, 48 h **(C)**, and 72 h **(D)**.

Spectral unmixing analysis was then applied to analyze the hyperspectral images. Before calculating the abundance of each pixel, VCA was used to extract the endmembers. The number of endmembers was set at 3 (the number of extracted principal components) plus or minus 1. Due to its simplicity, the number of endmembers for the healthy sample at 0 h was set at 2. In contrast, the sample at 72 h was more complicated, so the number of endmembers was set at 4. The extracted endmembers of each hyperspectral image are shown on the left in Figure 6. The endmembers with the same color were considered to characterize the same trait of the sample, because the similarity among them was high. At 0 h, the endmember 1 in Figure 6A represented the typical spectra of healthy leaf, and the endmember 2 with low reflectance in the NIR region characterized differences among regions due to sample heterogeneity among different parts of the leaf. The left part of the abundance maps in Figure 6A mostly contains pixels of endmember 1, while the right part is more relevant to endmember 2, since it shows the lower part of the barley leaf.

At 24 h, a new endmember was extracted (endmember 3) with higher reflectance in both visible and NIR regions, as seen in Figure 6B, which was consistent with the spectral characteristics of the light-yellow spots (Figure 3A). Therefore, endmember 3 could be interpreted as an indicator of light-yellow spots. As seen in Figure 6B, a number of bright points with high scores emerged in the abundance map of endmember 3 at 24 h. Therefore, the abundance maps based on spectral unmixing could distinguish lesion regions as early as 24 h. These identified infection spots could be validated by the subsequent images at 48 and 72 h. At 48 h, the extracted endmembers were similar to those at 24 h. The abundance map of endmember 1 reflected the distribution of pixels in the healthy leaf spectrum. The abundance map of endmember 3 showed clear disease spots marked by bright points (Figure 6C), which correspond to the light-yellow spots in the RGB image.

At 48 h, the light-yellow spots remained, but there was a significant growth in the area and the number of infected regions compared with 24 h. At 72 h, the infection had progressed significantly, and four endmembers were extracted to characterize the complex sample. Unlike in previous periods, the endmember that characterized differential regions of healthy leaf was no longer useful, and it was replaced by two new endmembers. In Figure 6D, endmember 2, with higher reflectance than endmember 3, indicates the emerging features of large yellow patches. As described earlier, the intensity of the reflectance spectra in the dark spot regions drastically dropped in the visible and NIR regions (Figure 3B).

An abundance map revealed that the dark spots observed in endmember 4 at 72 h (Figure 6D) developed from the yellow spots observed in endmember3 at 48 h (Figure 6C). In addition, as shown in Figure 6D, the abundance image of endmember 1 at 72 h still showed the presence of healthy regions, but they had shrunk in area. Meanwhile, the abundance map of endmember 3 still contained some light-yellow spots like in Figures 6B,C. However, compared with 48 h, the number of bright spots at 72 h in endmember 3 was decreased, since some of the disease spots had turned dark. Overall, the abundance images demonstrated the feasibility of spectral unmixing analysis for visualization of lesion regions, as the endmembers were more informative and easier to interpret than loading vectors of PCA, and the abundance maps returned clearer host–pathogen interactions and more details of the lesion sites.

**FIGURE 7:**
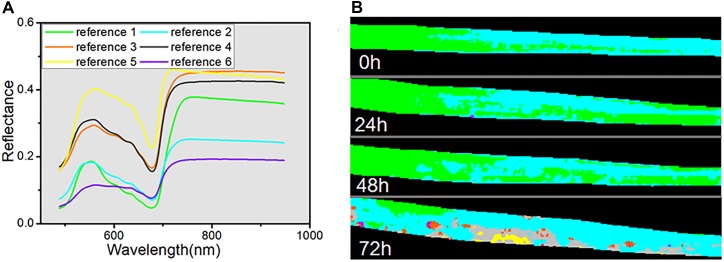
Reference spectra used for SAM **(A)** and color maps calculated by SAM **(B)**.

In addition, SAM, as a conventional spectral classification approach, was applied to compare its performance with spectral unmixing analysis. SAM classification is dependent on the pre-defined reference spectra, for which we used six endmembers extracted by VCA from four hyperspectral images. The classification results are shown as a color map in Figure 7B, and all the reference spectra are displayed in Figure 7A. Each pixel was set to the same color as its reference spectra. The classification maps indicated that, in the periods of 0, 24, and 48 h, the spectra of almost all the pixels were judged to represent normal healthy leaf (reference 1 with green color) or differential regions of the healthy leaf caused by differences of chemical composition (reference 2 with cyan color). Only a few pixels in the 24 h classification map were assigned to the reference spectra of dark spots (reference 6 with violet color). In contrast, the classification map at 72 h showed a greater variety, with all six categories represented. The region of the normal healthy leaf (green) was decreased to a small area, and the yellow color (reference 5) revealed the emergence of new yellow patches at 72 h. Meanwhile, the gray color (reference 4) revealed the light-gray transition regions surrounding the yellow regions, as seen in the RGB image (Figure 5A). The orange color (reference 3) marked the light-yellow spots, consistent with the abundance map of endmember 3 in Figure 6D, and the violet regions represented the dark spots. Nevertheless, the SAM classification of the hyperspectral images only provided clear visible symptoms at 72 h.

## Discussion

The first 72 h after inoculation is the crucial stage of disease development. The newly formed infection hyphae primarily attacks the epidermal cells. With the progress of infection, cell necrosis occurs, characterized by an abundance of collapsed mesophyll cells (Ulferts et al., 2015). In addition, the intracellular pigment is broken down. These collapsed cells, with decomposed pigment, accumulate into visible lesion tissues over a relatively long infection time. Therefore, it is necessary to identify the infectious degree and visualize the disease lesions early, before visual symptoms appear. Traditional molecular methods like PCR, have intricate sample preparation and low sensitivity, while recently newer methods using real-time quantitative PCR can improve the speed and accuracy of *M. oryzae* detection using molecular techniques (Sun et al., 2015). Bini et al. (2018) also develop a qPCR assay for the early detection of the eucalyptus rust disease. However, it requires destructive sampling and does not provide additional phenotyping information or allow *in situ* analysis. Various studies have reported that HSI is a promising technique for non-invasive and *in situ* measurement of foliar disease, and could achieve the separation between healthy and infected samples (Zhang et al., 2012; Xie et al., 2017). While, in our study, the optimized variables acquired from β of PLSR were distributed mainly in the regions of 490–550 nm and 900–950 nm (as seen in Figure 4). The reflectance at 490–550 nm is related to the green appearance of the leaves, and that at 900–950 nm represents the changes of tissue structure and components (Kuska et al., 2015). Compared with β of PLSR, CARS returned better variables for classification as Supplementary Table S1 described. As for CARS, the optimized variables were mainly concentrated in the regions of 600–650 nm and 750–950 nm. The reflectance at 600–650 nm corresponds to the red and orange regions of the spectrum, and is closely related to the absorption of light by chlorophyll within the leaves. The reflectance at 750–950 nm contained extensive information on tissue structure and chemical composition. Therefore, the attempt at sample classification using these optimized variables was based on the content of pigments and information about tissue structure and chemical composition. Designing an effective criterion to extract an optimal subset of spectral wavelengths with fact meanings is hard for data classification. We found that changes of pigments content and tissue structure could be determined by hyperspectral data analysis earlier than it would be visible to the human eye.

In addition to early detection, the visualization of lesion regions is equally important. Commonly used machine vision recognition mainly depends on visual symptoms. Following segmentation of the leaf from the background, the major features such as color and texture were extracted and used as inputs for machine learning (Zhou et al., 2015; Barbedo et al., 2016). Recently, deep learning methods have been applied to analyze images with leaf lesions. Ghosal et al. (2018) demonstrated a deep learning method to detect foliar plant stresses in soybean using images of visual symptoms. However, some infection symptoms could not be clearly discerned from the RGB images (Figure 2B), nor could early detection be achieved by image processing. In contrast, the hyperspectral images contained much more information than the RGB images, but the averaged information obscured many of the spectral characteristics in the lesion areas. Therefore, effective methods were required to mine and visualize the useful portion of this information. Moreover, consecutive scanning enabled us to track the progress of infection effectively and trace the lesion location in the early stages of the foliar disease.

Traditional methods to visualize the change of lesion regions were manual marking (Leucker et al., 2015), SAM classification (Mahlein et al., 2012a) and PCA score images (Williams et al., 2012). Manual marking is according to the visible spots, which is not suitable for automatic detection in the early stage of infection. SAM classification is dependent on the pre-defined reference spectra, which needs a priori knowledge. Additionally, as Figure 7 indicated, the SAM classification of the hyperspectral images only provided clear symptoms at 72 h, and performed poorly at the earlier infection times. Since the spectral response of different pixels in leaves reflects very complex information, we cannot simply identify one pixel into a certain class.

The results also proved that SAM classification is inaccurate. By contrast, the PCA-based images demonstrated the feasibility for early identification, as the score map of PC3 at 24 h (as seen in Figure 5C) showed a recognizable image of the lesion regions. Nevertheless, the PCA-based images were relatively hard to interpret, as the loading vectors obtained from the calculation contained both positive and negative values. In addition, the information expressed by the score maps at 72 h was not complete enough to allow discrimination. Due to the drawbacks of PCA-based imaging, we introduced the technique of abundance imaging, based on the theory of spectral unmixing analysis. VCA can select the most representative pixels of all pixels as endmembers, which ensures the meaning of the extracted endmembers. Meanwhile, the abundance maps reflect the optimal ratio of each endmember, which show more detailed information on lesion regions. As seen in Figure 6B, the abundance maps of endmember 3 could distinguish lesion regions as early as 24 h and, more importantly, the endmember 3 represented the spectral response of light-yellow spots (as seen in Figure 3A). Thus, changes in endmember spectra can be used to mark disease progression. In stressed plants, chlorophyll tends to decline more rapidly than other pigments such as carotenoids (Merzlyak et al., 1999). As a result of reduced absorption by pigments, reflectance at visible wavelengths (especially 550–650 nm) increased consistently in infected leaves (comparing endmembers 1 and 3, Figure 6). Similar changes in reflectance have been previously reported (Carter, 1993). After infection, the organizational integrity of tissue changed significantly (Supplementary Figure S1); such changes in internal tissue structure can cause increases in the NIR region from 700 nm to 1000 (Sinclair et al., 1971). As infection gradually progressed outward from the infection site, extensive pigment degradation and tissue damage resulted in loss of reflectance in both visible and NIR regions, as seen in endmember 4 (Figure 6D). Thus, disease progression can be observed by a pattern of first rising and then falling of the reflectance spectra. The abundance images demonstrated the feasibility of spectral unmixing analysis for visualization of lesion regions, as the endmembers were more informative and easier to interpret, and the abundance maps returned clearer host-pathogen interactions and more details of the lesion sites.

Overall, SAM could not achieve early detection, and both PCA and spectral unmixing could identify the lesion regions at 24 h. However, the PCA loading vectors were harder to interpret than the endmembers of spectral unmixing. Furthermore, the abundance maps expressed much more detail of lesion type and distribution than the score maps. We introduced the spectral unmixing theory from hyperspectral remote sensing to solve the obtained hyperspectral images, which achieved new insight into better interpretation of the original data and clearer visualization of host-pathogen interactions. Since this study was conducted under laboratory conditions, there are still further important points to note in order to apply this method in the field. The throughput of phenotyping would depend on the scanning speed. Normally a push broom device may take a long time to acquire one image, if the spectral and spatial resolutions are high. A snapshot approach may better adapt to this condition (Lowe et al., 2017). Since spectral unmixing needs to extract feature information from the entire image, it is necessary to put a complete hyperspectral image into the calculation rather than calculate a part of data while scanning. Moreover, as the number of pixels increases, the computational cost of feature extraction needs to be considered. One possible solution is searching the possible endmembers in advance through some pre-experiments. However, these are also common problems when large amounts of data are analyzed with other methods. The advantages of spectral unmixing could help better visualize the disease phenotype.

## Conclusion

This research analyzed the characteristic symptoms and development of barley leaves infected by *M. oryzae* (Guy11) using HSI. The earliest symptom was the appearance of light-yellow spots, with high spectral reflectance, at 48 h. Some of the light-yellow spots later turned dark, with low spectral reflectance. The averaged spectra of all the leaves were used to identify the infection periods and distinguish infected samples at 24 h. CARS was performed to select the 30 optimal (most informative) wavelengths, related to pigments and tissue structure, which simplified the model and improved the interpretability. The combination of CARS and LDA demonstrated the best performance, with 100 and 98.1% classification accuracies of the calibration and prediction sets, respectively, which demonstrated the feasibility of HSI for early *M. oryzae* disease detection. Importantly, the identification of early lesion regions was possible by applying various chemometric methods to the HSI images. The results indicated that the abundance maps calculated by spectral unmixing analysis could unambiguously reveal the disease regions even at 24 h, and clearly showed the complex lesion distribution of the leaf at 72 h. Moreover, this method allowed easier interpretation than the other tested methods. Taken together, these results demonstrated the effectiveness of HSI combined with data analysis for early disease detection, tracking and recognition of lesion regions. This technology enables the high-throughput screening of early fungal disease on plant leaves.

## Author Contributions

R-QZ, X-LL, and Q-ML designed the experiments. R-QZ, J-JJ, and Z-ZS performed the experiments. R-QZ, J-JJ, X-LL, and X-JY contributed to the data analysis and wrote the manuscript. YH, YT, and S-ML provided suggestions on the experiment design and discussion sections.

## Conflict of Interest Statement

The authors declare that the research was conducted in the absence of any commercial or financial relationships that could be construed as a potential conflict of interest.
