# Supplementary material for: Early Detection of Magnaporthe oryzae-Infected Barley Leaves and Lesion Visualization Based on Hyperspectral Imaging
Source: Front Plant Sci. 2019 Jan 15;9:1962. doi: 10.3389/fpls.2018.01962 (PMC6341029; doi:10.3389/fpls.2018.01962)

Supplementary Material

Early Detection of Magnaporthe oryzae-infected Barley Leaves and Lesion Location Based on Hyperspectral Imaging

Rui-Qing Zhou, Juan-Juan Jin, Zhen-Zhu Su, Ning Xu, Yu Tang, Shaoming Tang, Yong He*, Xiao-Li Li*

*** Correspondence:** Xiao-Li Li: [xiaolili@zju.edu.cn](mailto:xiaolili@zju.edu.cn)

Yong He: [yhe@zju.edu.cn](mailto:yhe@zju.edu.cn)

# Supplementary Figures

To describe the microscopic structures of lesion locations during the infection process, three typical samples were embedded in paraffin. 5μm paraffin-embedded sections were stained by periodic acid–Schiff staining after dewaxing. Microscopic images were obtained by an optical microscope (ICC50W, Leica, Germany)


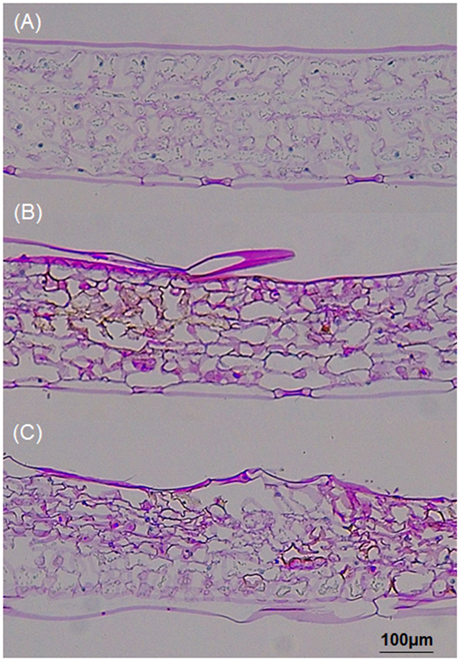


**Figure S1.** Microscopic images of stained sections corresponding to healthy tissue (A), light-yellow spots (B) and dark spots (C).

The receiver operating characteristic (ROC) curves of the best model based on LDA and the optimal variables obtained by CARS were calculated. The area under the ROC curves of all four periods are very high, which prove that the model has high classification accuracy.


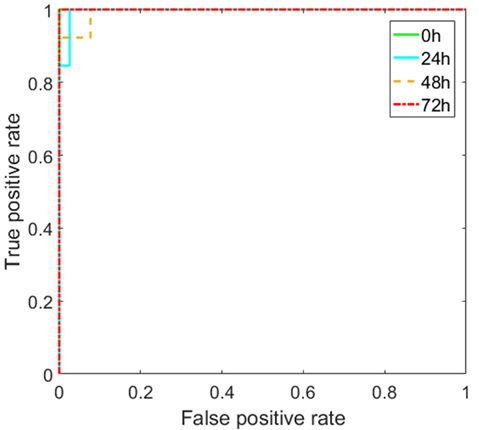


**Figure S2.** ROC curve of the best model based on LDA and the optimal variables obtained by CARS.

# Supplementary Tables

**Table S1.** Results of classification models using optimal variables

| Method | Regression coefficient of PLS | | CARS | |
| --- | --- | --- | --- | --- |
|  | Calibration set (%) | Prediction set (%) | Calibration set (%) | Prediction set (%) |
| LDA | 98 | 92.3 | 100 | 98.1 |
| PLS-DA | 75 | 71.2 | 98 | 76.9 |
| KNN | 67 | 67.3 | 74 | 59.6 |
| SIMCA | 83 | 73.1 | 97 | 82.7 |

# Supplementary graphic for table of contents


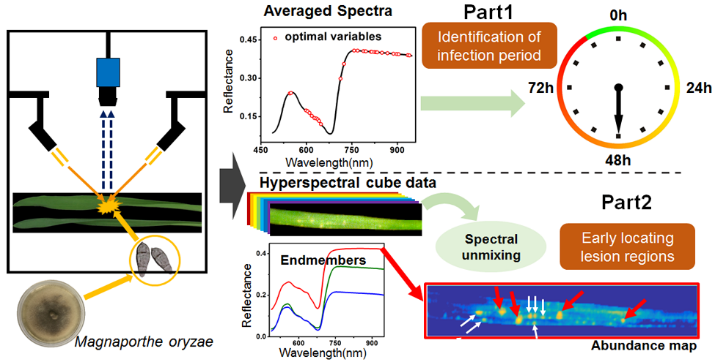

Supplement: Supplementary file 1 [file Data_Sheet_1.docx]
